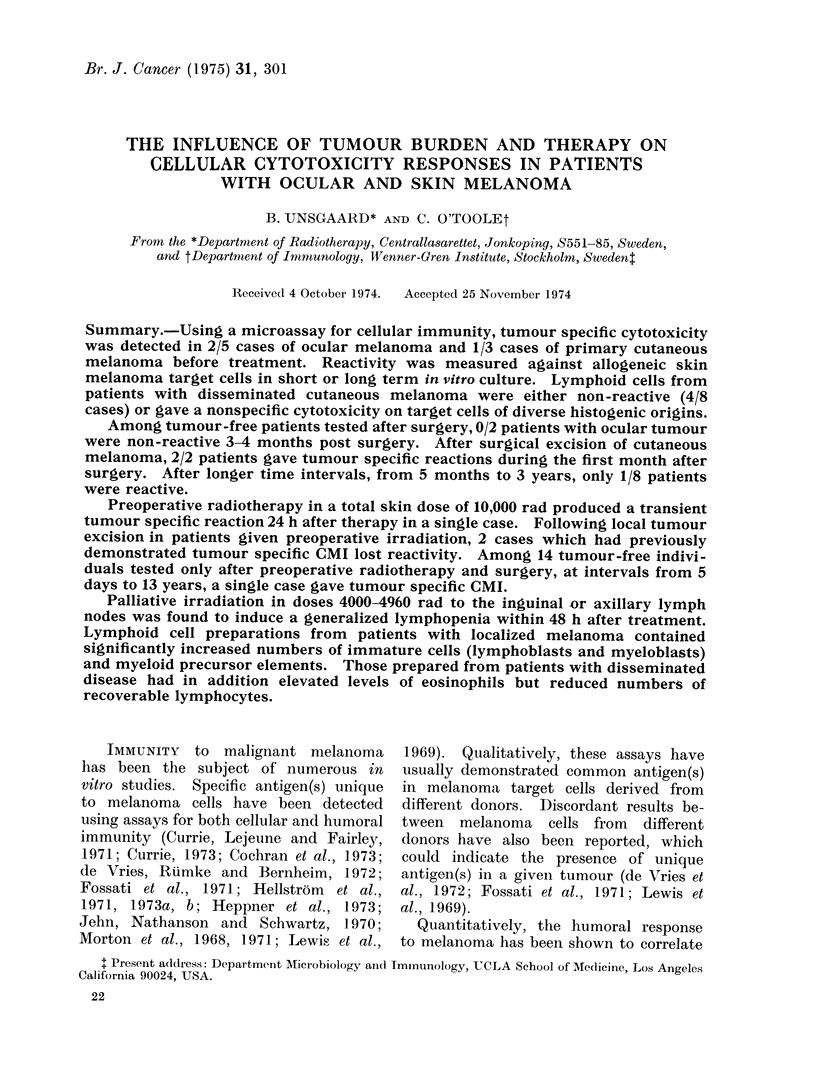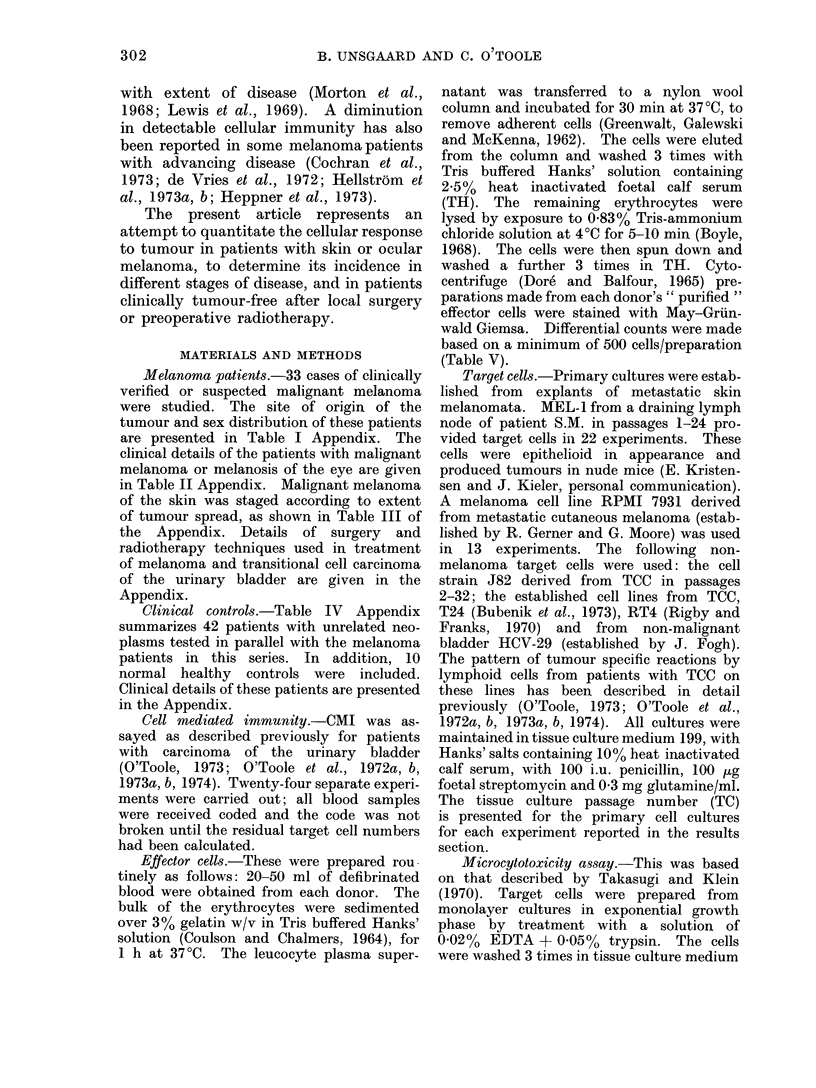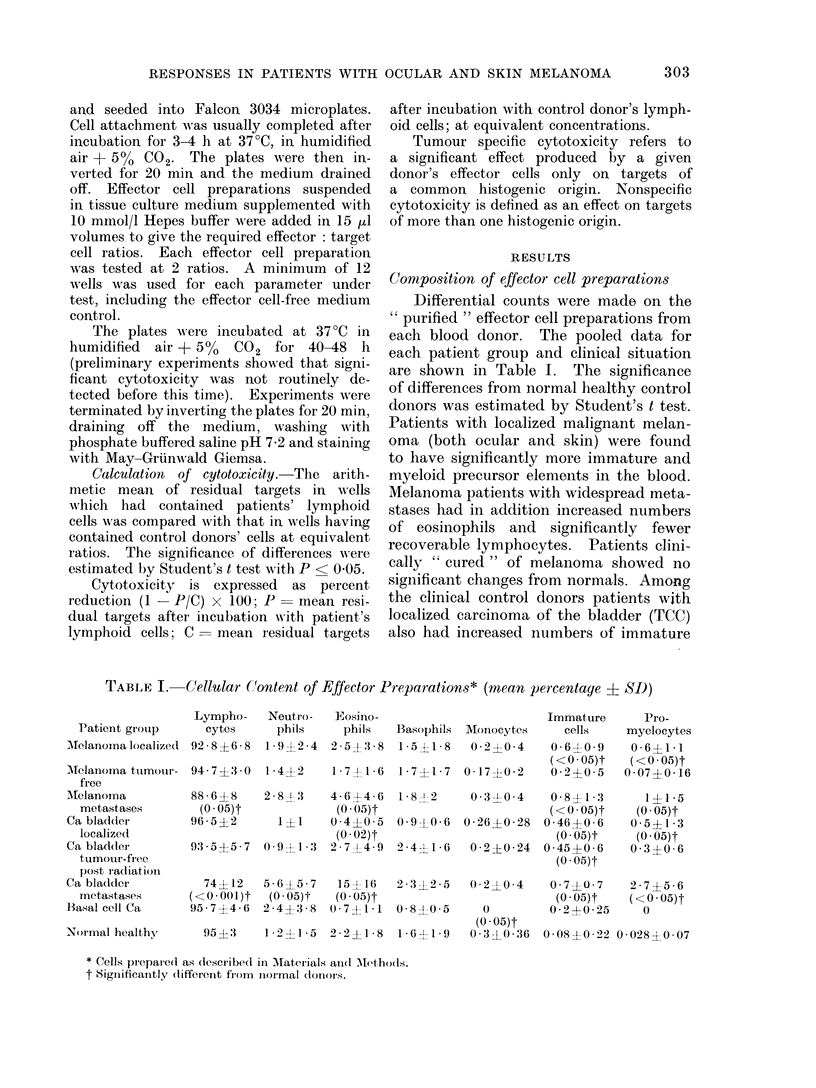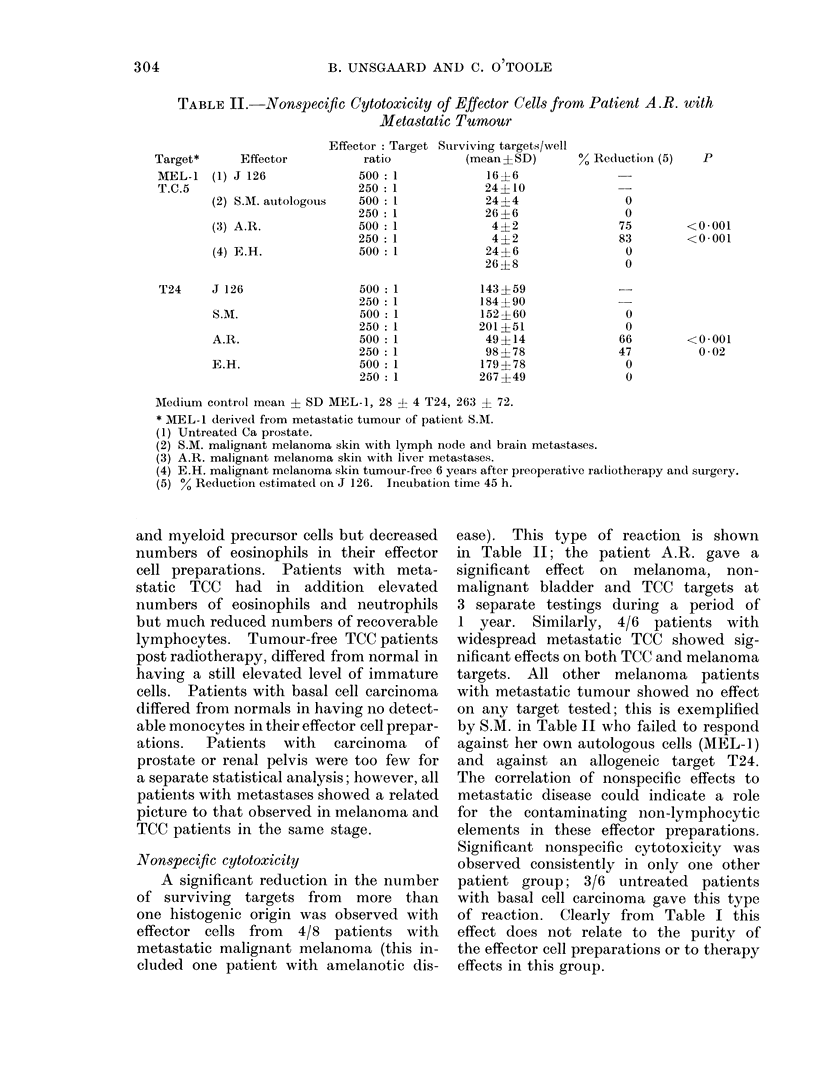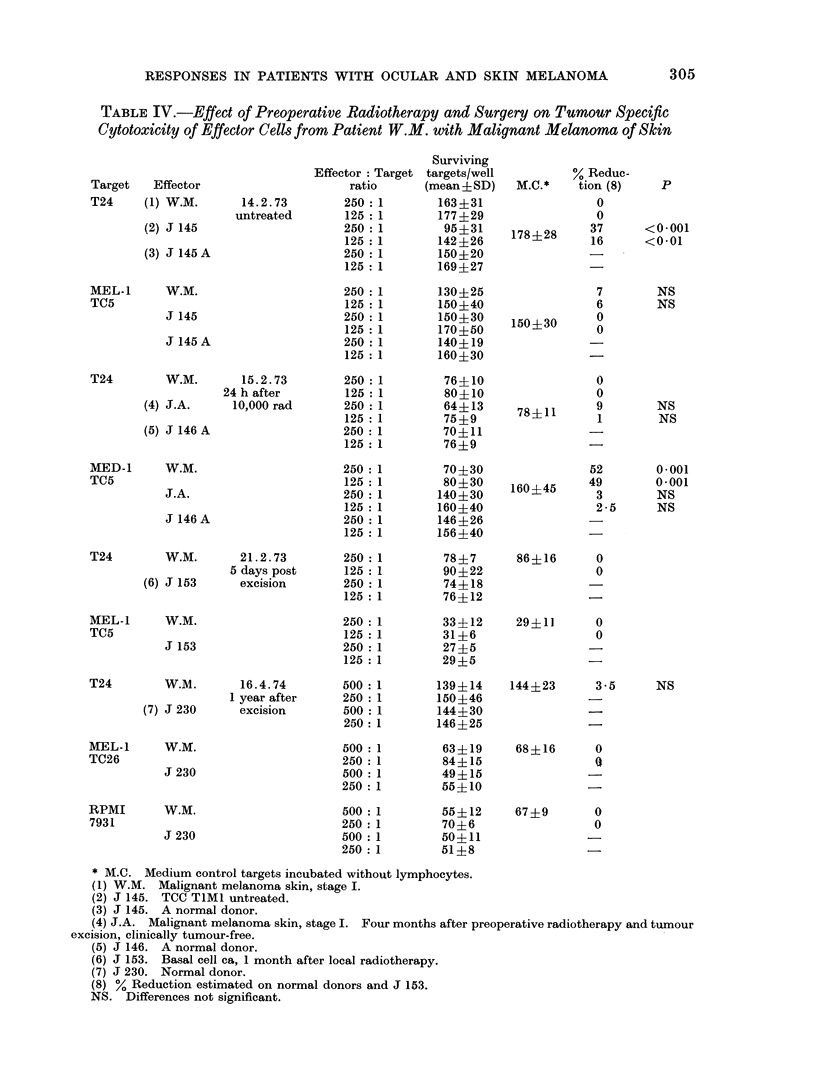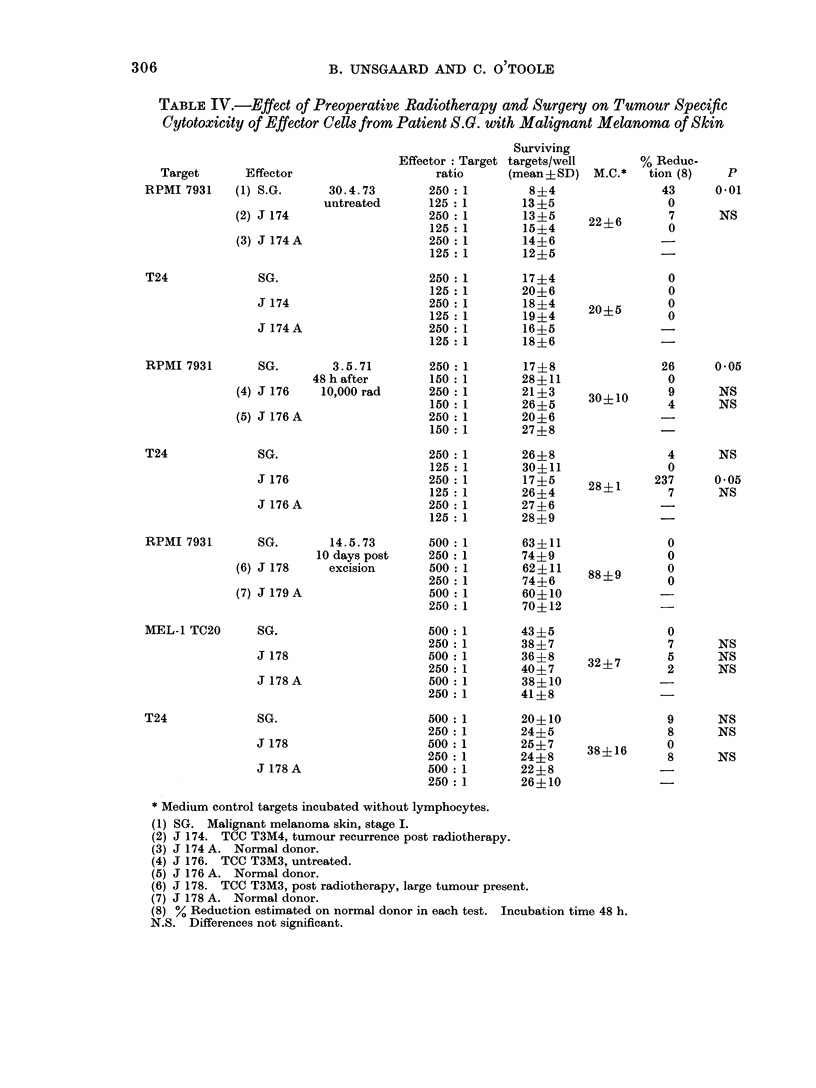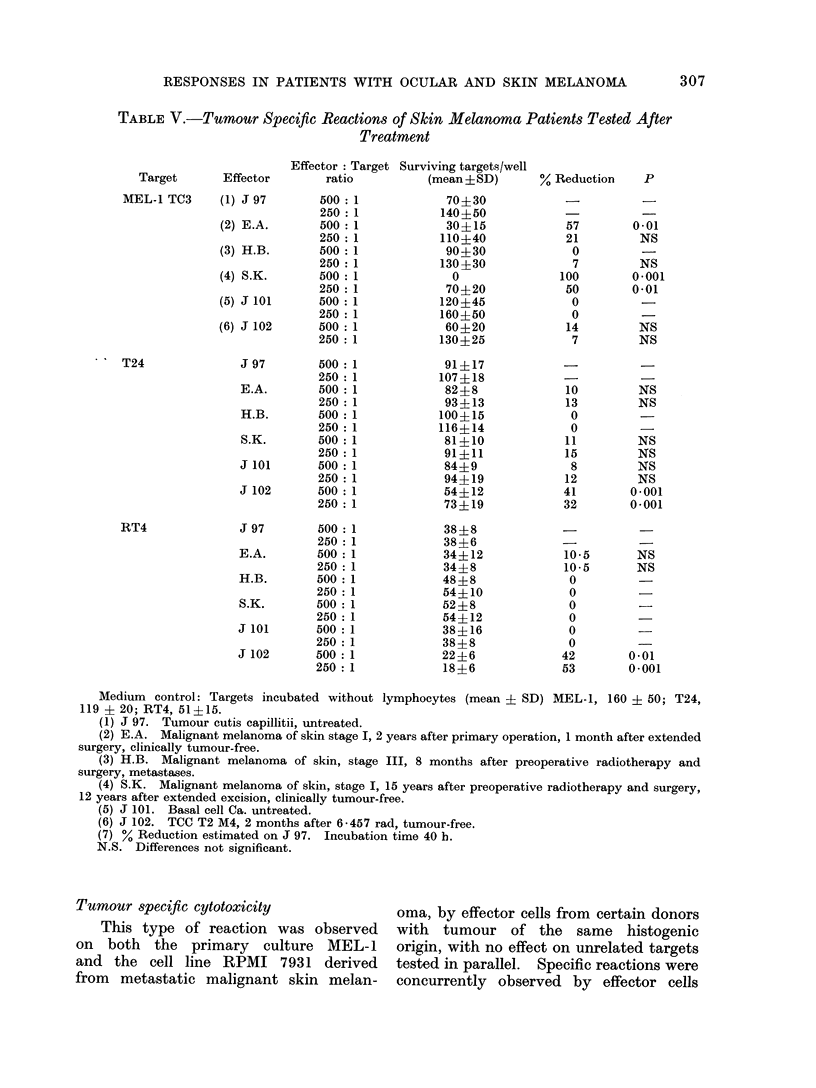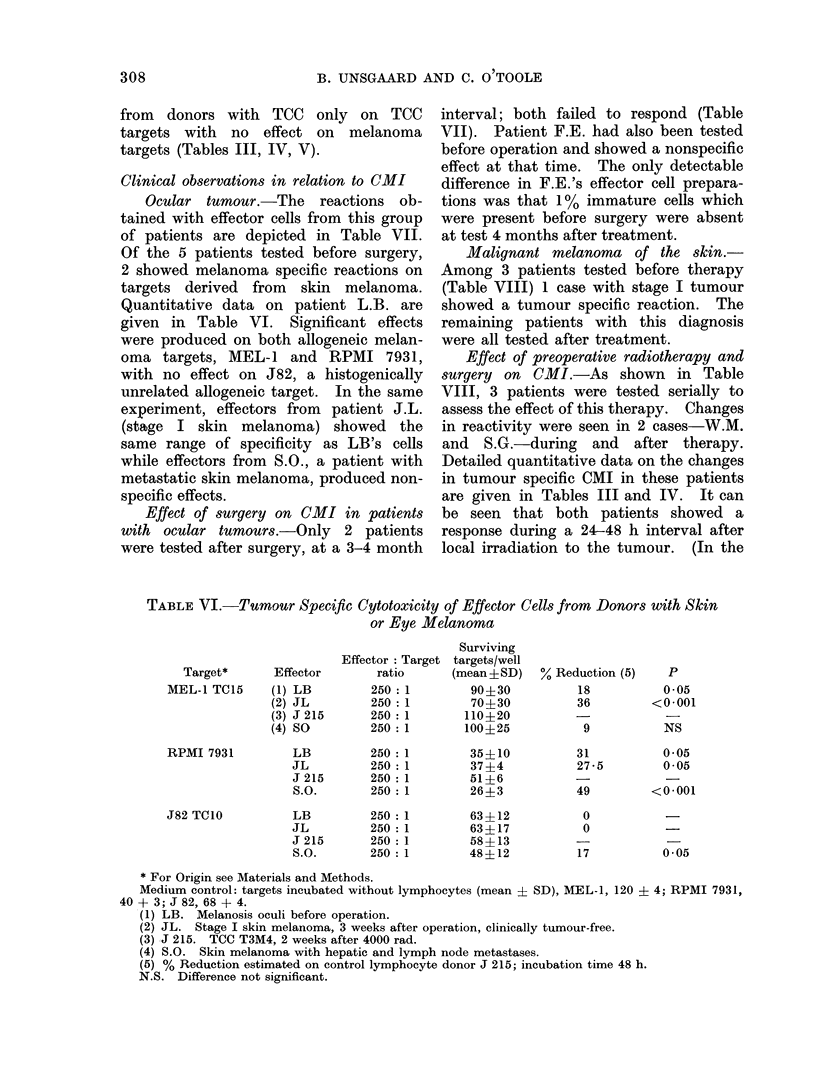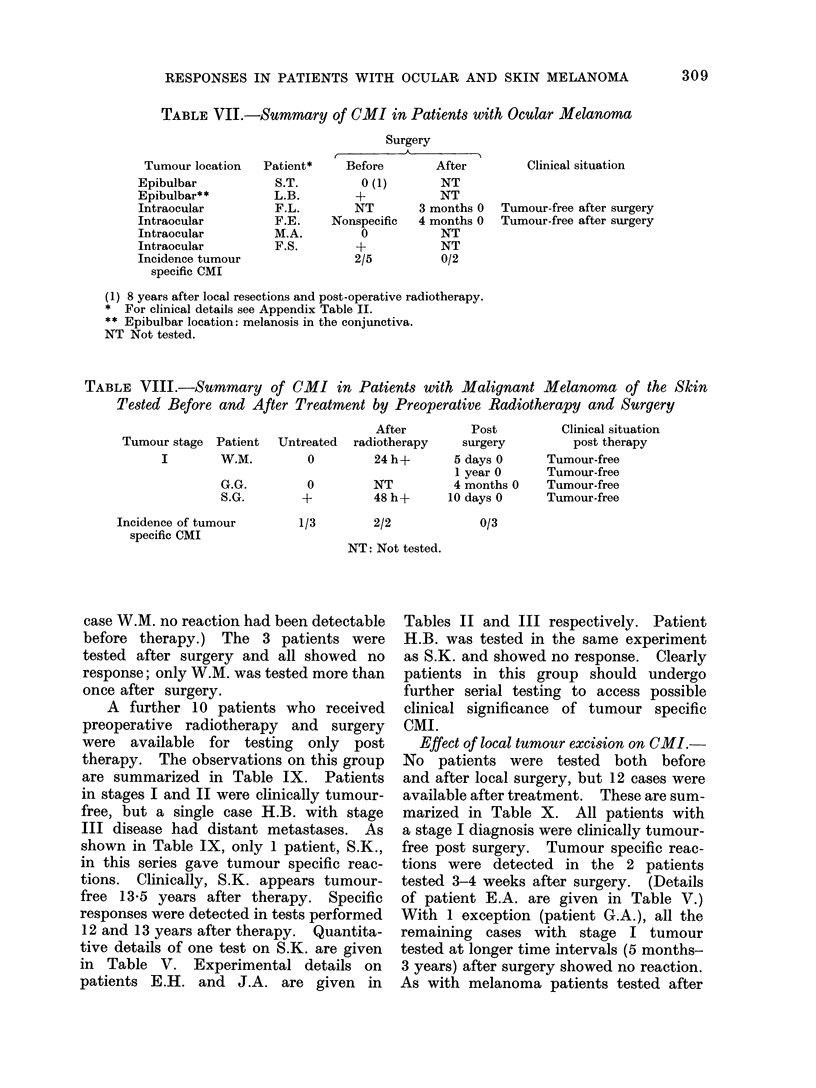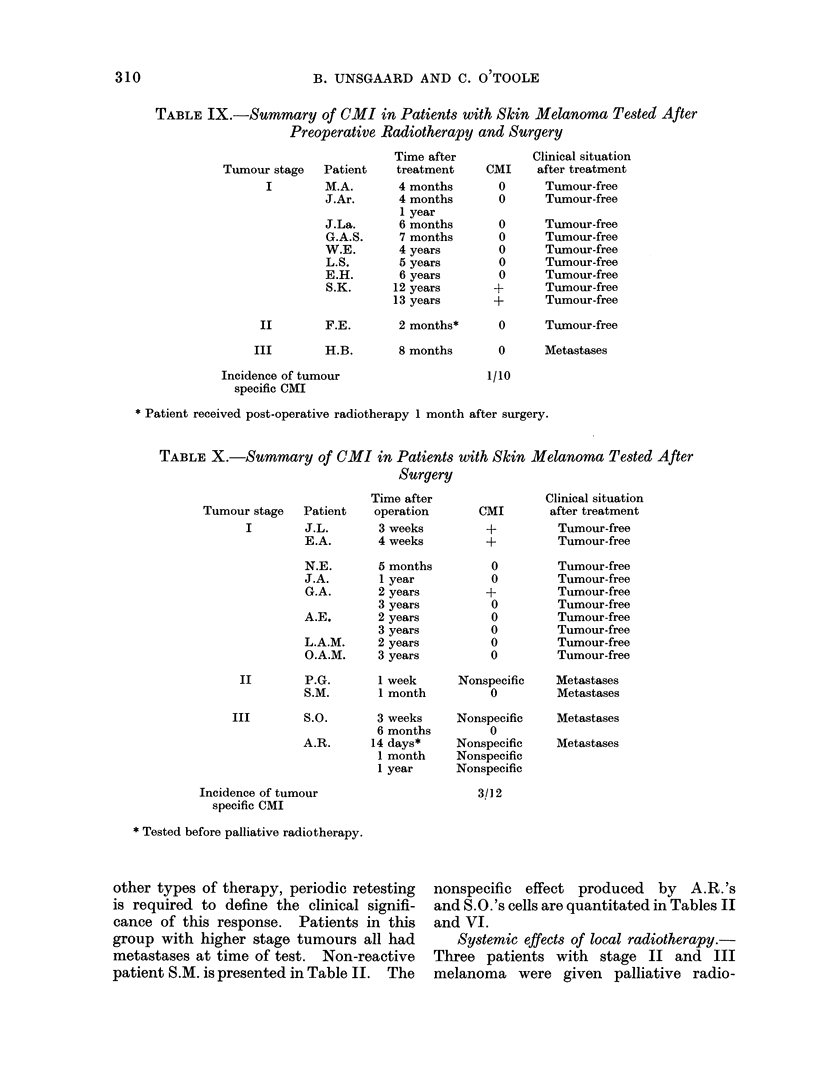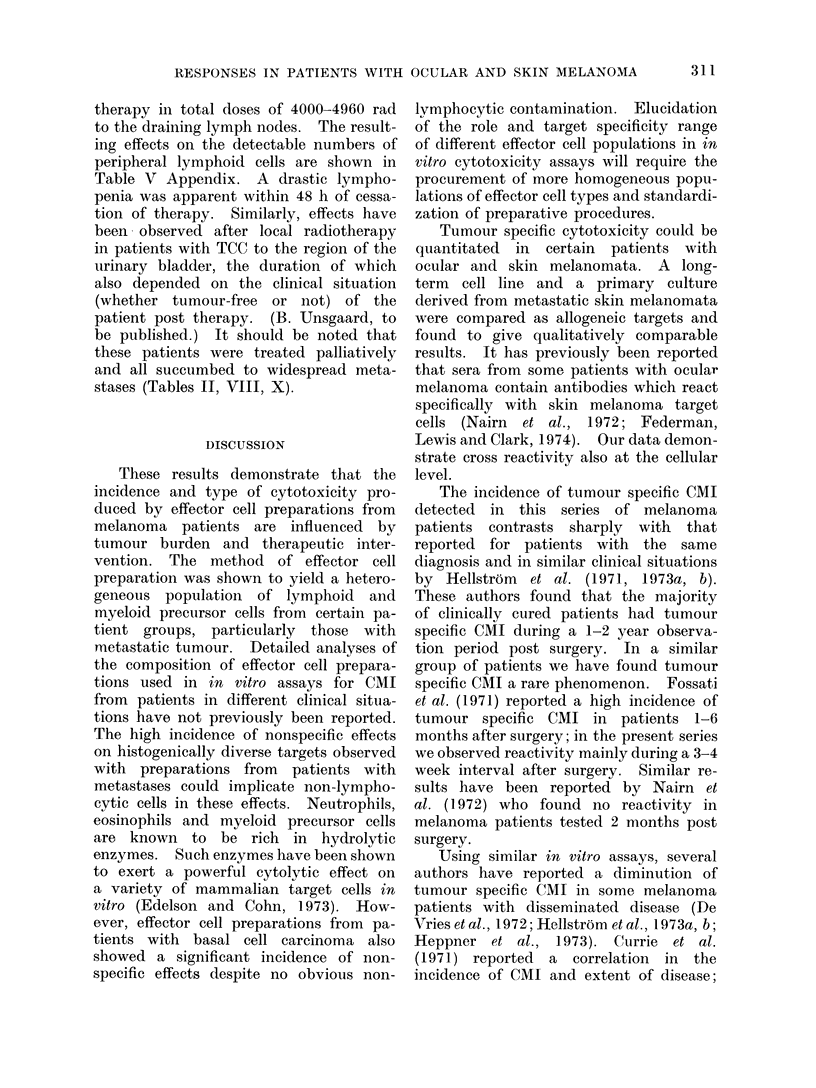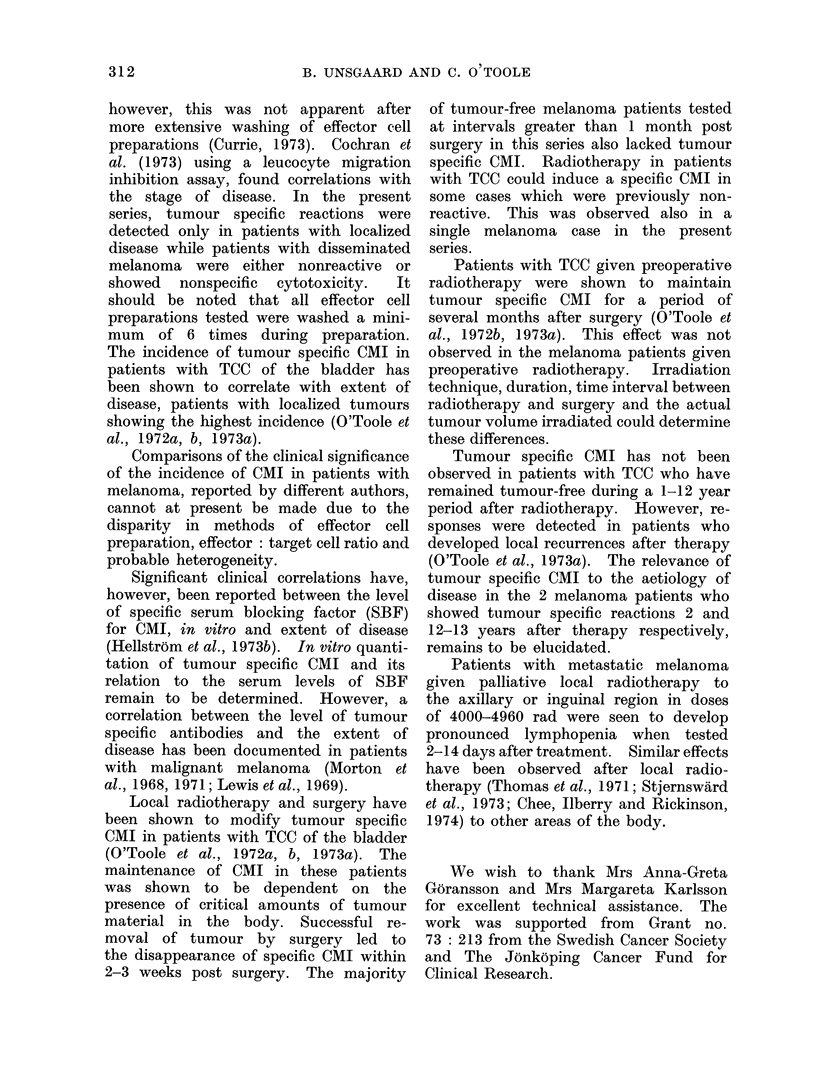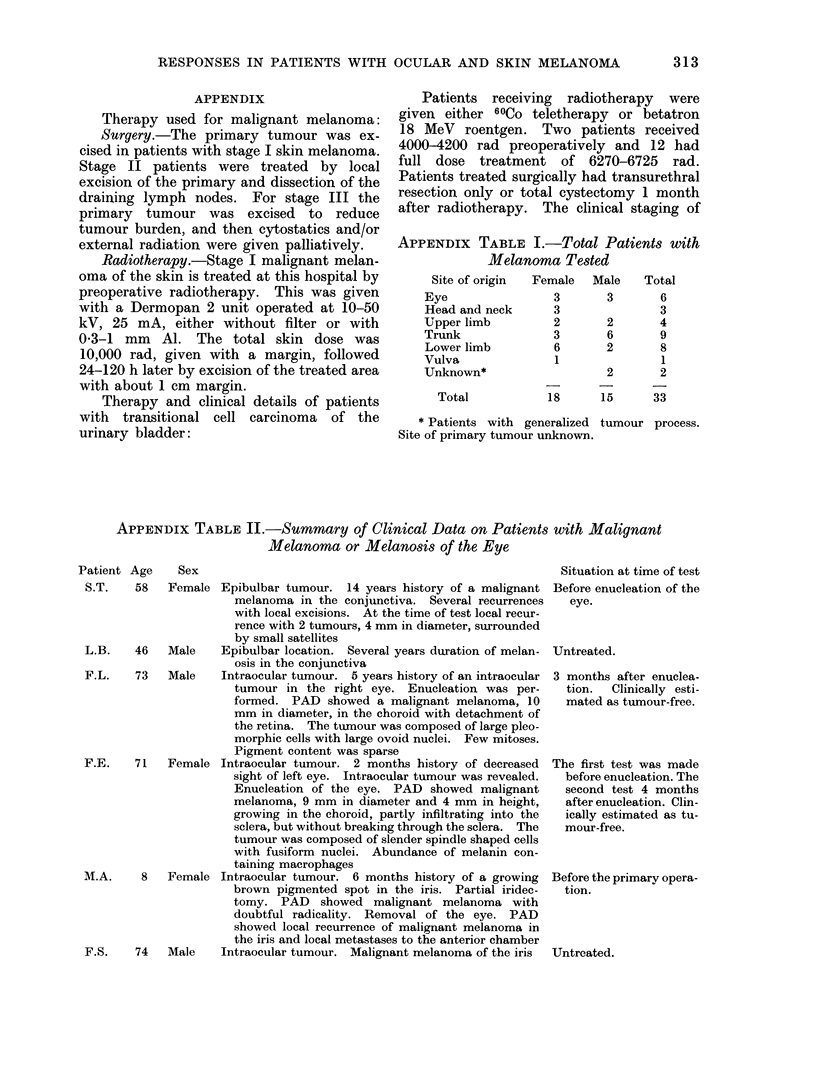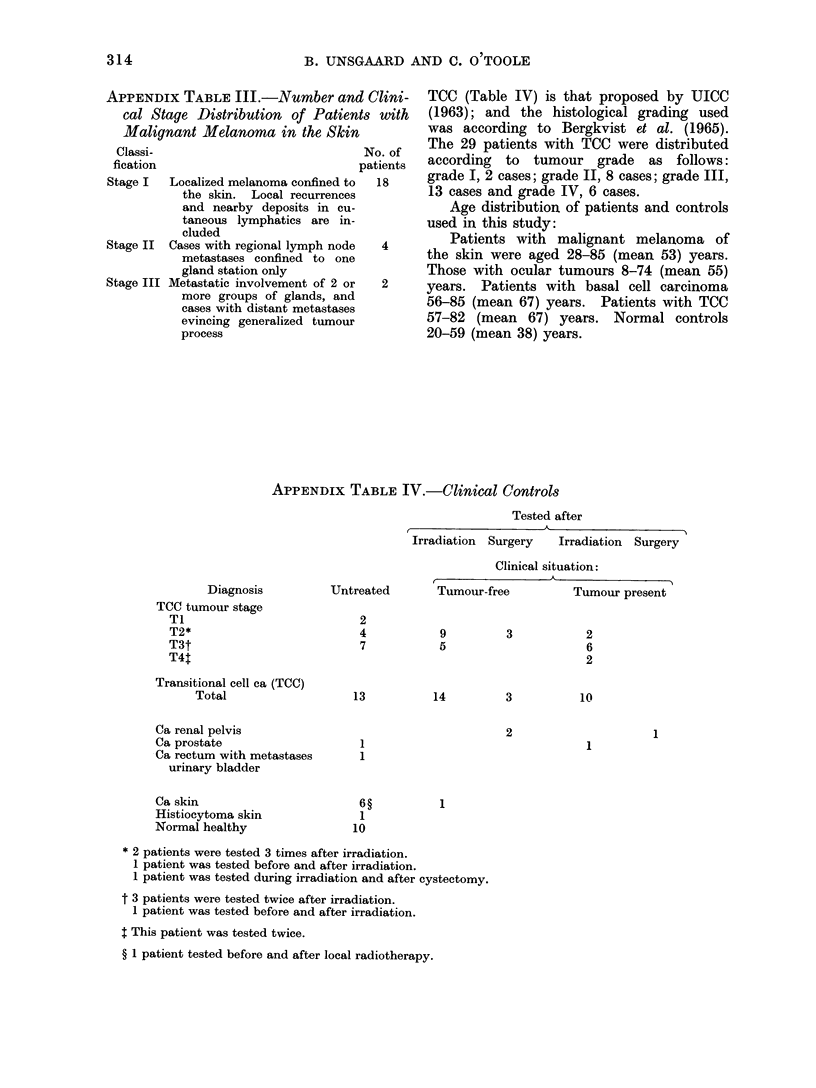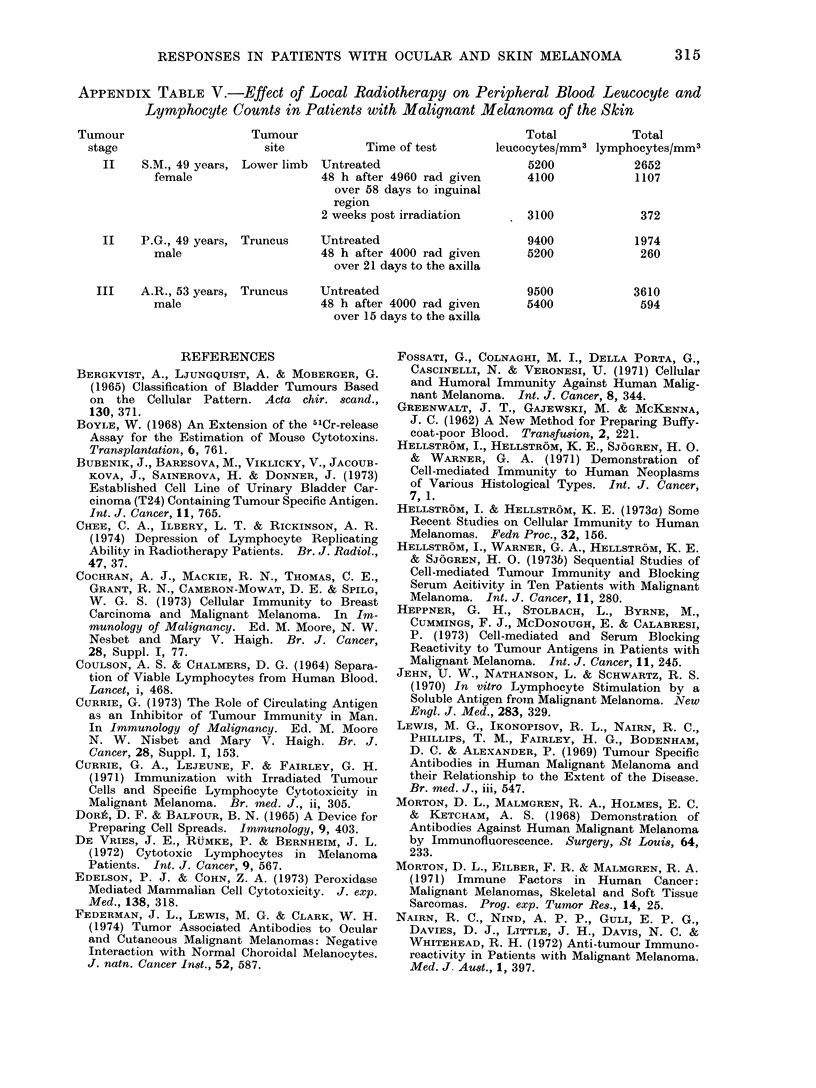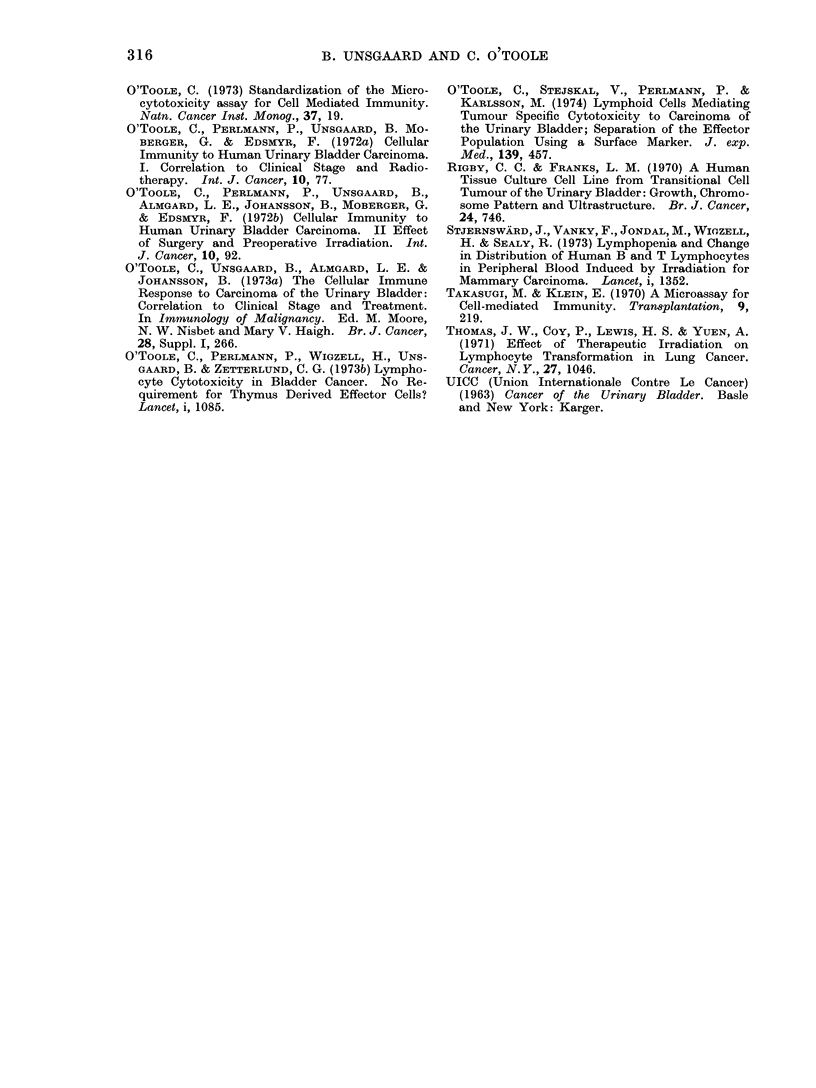# The influence of tumour burden and therapy on cellular cytotoxicity responses in patients with ocular and skin melanoma.

**DOI:** 10.1038/bjc.1975.65

**Published:** 1975-03

**Authors:** B. Unsgaard, C. O'Toole

## Abstract

Using a microassay for cellular immunity, tumour specific cytotoxicity was detected in 2/5 cases of ocular melanoma and 1/3 cases of primary cutaneous melanoma before treatment. Reactivity was measured against allogeneic skin melanoma target cells in short or long term in vitro culture. Lymphoid cells from patients with disseminated cutaneous melanoma were either non-reactive (4/8 cases) or gave a nonspecific cytotoxicity on target cells of diverse histogenic origins. Among tumour-free patients tested after surgery, 0/2 patients with ocular tumour were non-reactive 3-4 months post surgery. After sugical excision of cutaneous melanoma 2/2 patients gave tumour specific reactions during the first month after surgery. After longer time intervals, from 5 months to 3 years, only 1/8 patients were reactive. Preoperative radiotherapy in a total skin dose of 10,000 rad produ-ed a transient tumour specific reaction 24 h after therapy in a single case. Following local tumour excision in patients given preoperative irradiation, 2 cases which had previously demonstrated tumour specific CMI lost reactivity. Among 14 tumour-free individuals tested only after preoperative radiotherapy and surgery, at intervals from 5 day to 13 years, a single case gave tumour specific CMI. Palliative irradiation in doses 4000-4960 rad to the inguinal or axillary lymph nodes was found to induce a generalized lymphopenia within 48 h after treatment. Lymphoid cell preparations from patients with localized melanoma contained significantly increased numbers of immature cells (lymphoblasts and myeloblasts) and myeloid precursor elements. Those prepared from patients with disseminated disease had in addition elevated levels of eosinophils but reduced numbers of recoverable lymphocytes.


*Br. J. Cancer* (1975) **31**, 301

### THE INFLUENCE OF TUMOUR BURDEN AND THERAPY ON CELLULAR CYTOTOXICITY RESPONSES IN PATIENTS WITH OCULAR AND SKIN MELANOMA

B. UNSGAARD* AND C. O'TOOLE†

*From the *Department of Radiotherapy, Centrallasarettet, Jonkoping, S551–85, Sweden, and †Department of Immunology, Wenner-Gren Institute, Stockholm, Sweden‡*

Received 4 October 1974. Accepted 25 November 1974

**Summary.—Using a microassay for cellular immunity, tumour specific cytotoxicity was detected in 2/5 cases of ocular melanoma and 1/3 cases of primary cutaneous melanoma before treatment. Reactivity was measured against allogeneic skin melanoma target cells in short or long term *in vitro* culture. Lymphoid cells from patients with disseminated cutaneous melanoma were either non-reactive (4/8 cases) or gave a nonspecific cytotoxicity on target cells of diverse histogenic origins.**

**Among tumour-free patients tested after surgery, 0/2 patients with ocular tumour were non-reactive 3–4 months post surgery. After surgical excision of cutaneous melanoma, 2/2 patients gave tumour specific reactions during the first month after surgery. After longer time intervals, from 5 months to 3 years, only 1/8 patients were reactive.**

**Preoperative radiotherapy in a total skin dose of 10,000 rad produced a transient tumour specific reaction 24 h after therapy in a single case. Following local tumour excision in patients given preoperative irradiation, 2 cases which had previously demonstrated tumour specific CMI lost reactivity. Among 14 tumour-free individuals tested only after preoperative radiotherapy and surgery, at intervals from 5 days to 13 years, a single case gave tumour specific CMI.**

**Palliative irradiation in doses 4000–4960 rad to the inguinal or axillary lymph nodes was found to induce a generalized lymphopenia within 48 h after treatment. Lymphoid cell preparations from patients with localized melanoma contained significantly increased numbers of immature cells (lymphoblasts and myeloblasts) and myeloid precursor elements. Those prepared from patients with disseminated disease had in addition elevated levels of eosinophils but reduced numbers of recoverable lymphocytes.**

IMMUNITY to malignant melanoma has been the subject of numerous *in vitro* studies. Specific antigen(s) unique to melanoma cells have been detected using assays for both cellular and humoral immunity (Currie, Lejeune and Fairley, 1971; Currie, 1973; Cochran *et al.*, 1973; de Vries, Rümke and Bernheim, 1972; Fossati *et al.*, 1971; Hellström *et al.*, 1971, 1973*a*, *b*; Heppner *et al.*, 1973; Jehn, Nathanson and Schwartz, 1970; Morton *et al.*, 1968, 1971; Lewis *et al.*, 1969). Qualitatively, these assays have usually demonstrated common antigen(s) in melanoma target cells derived from different donors. Discordant results between melanoma cells from different donors have also been reported, which could indicate the presence of unique antigen(s) in a given tumour (de Vries *et al.*, 1972; Fossati *et al.*, 1971; Lewis *et al.*, 1969).

Quantitatively, the humoral response to melanoma has been shown to correlate

‡ Present address: Department Microbiology and Immunology, UCLA School of Medicine, Los Angeles California 90024, USA.

with extent of disease (Morton *et al.*, 1968; Lewis *et al.*, 1969). A diminution in detectable cellular immunity has also been reported in some melanoma patients with advancing disease (Cochran *et al.*, 1973; de Vries *et al.*, 1972; Hellström *et al.*, 1973*a*, *b*; Heppner *et al.*, 1973).

The present article represents an attempt to quantitate the cellular response to tumour in patients with skin or ocular melanoma, to determine its incidence in different stages of disease, and in patients clinically tumour-free after local surgery or preoperative radiotherapy.

### MATERIALS AND METHODS

*Melanoma patients.*—33 cases of clinically verified or suspected malignant melanoma were studied. The site of origin of the tumour and sex distribution of these patients are presented in Table I Appendix. The clinical details of the patients with malignant melanoma or melanosis of the eye are given in Table II Appendix. Malignant melanoma of the skin was staged according to extent of tumour spread, as shown in Table III of the Appendix. Details of surgery and radiotherapy techniques used in treatment of melanoma and transitional cell carcinoma of the urinary bladder are given in the Appendix.

*Clinical controls.*—Table IV Appendix summarizes 42 patients with unrelated neoplasms tested in parallel with the melanoma patients in this series. In addition, 10 normal healthy controls were included. Clinical details of these patients are presented in the Appendix.

*Cell mediated immunity.*—CMI was assayed as described previously for patients with carcinoma of the urinary bladder (O'Toole, 1973; O'Toole *et al.*, 1972*a*, *b*, 1973*a*, *b*, 1974). Twenty-four separate experiments were carried out; all blood samples were received coded and the code was not broken until the residual target cell numbers had been calculated.

*Effector cells.*—These were prepared routinely as follows: 20–50 ml of defibrinated blood were obtained from each donor. The bulk of the erythrocytes were sedimented over 3% gelatin w/v in Tris buffered Hanks' solution (Coulson and Chalmers, 1964), for 1 h at 37 °C. The leucocyte plasma supernatant was transferred to a nylon wool column and incubated for 30 min at 37 °C, to remove adherent cells (Greenwalt, Galewski and McKenna, 1962). The cells were eluted from the column and washed 3 times with Tris buffered Hanks' solution containing 2·5% heat inactivated foetal calf serum (TH). The remaining erythrocytes were lysed by exposure to 0·83% Tris-ammonium chloride solution at 4 °C for 5–10 min (Boyle, 1968). The cells were then spun down and washed a further 3 times in TH. Cytocentrifuge (Doré and Balfour, 1965) preparations made from each donor's " purified " effector cells were stained with May-Grünwald Giemsa. Differential counts were made based on a minimum of 500 cells/preparation (Table V).

*Target cells.*—Primary cultures were established from explants of metastatic skin melanomata. MEL-1 from a draining lymph node of patient S.M. in passages 1–24 provided target cells in 22 experiments. These cells were epithelioid in appearance and produced tumours in nude mice (E. Kristensen and J. Kieler, personal communication). A melanoma cell line RPMI 7931 derived from metastatic cutaneous melanoma (established by R. Gerner and G. Moore) was used in 13 experiments. The following non-melanoma target cells were used: the cell strain J82 derived from TCC in passages 2–32; the established cell lines from TCC, T24 (Bubenik *et al.*, 1973), RT4 (Rigby and Franks, 1970) and from non-malignant bladder HCV-29 (established by J. Fogh). The pattern of tumour specific reactions by lymphoid cells from patients with TCC on these lines has been described in detail previously (O'Toole, 1973; O'Toole *et al.*, 1972*a*, *b*, 1973*a*, *b*, 1974). All cultures were maintained in tissue culture medium 199, with Hanks' salts containing 10% heat inactivated calf serum, with 100 i.u. penicillin, 100 $\mu$g foetal streptomycin and 0·3 mg glutamine/ml. The tissue culture passage number (TC) is presented for the primary cell cultures for each experiment reported in the results section.

*Microcytotoxicity assay.*—This was based on that described by Takasugi and Klein (1970). Target cells were prepared from monolayer cultures in exponential growth phase by treatment with a solution of 0·02% EDTA + 0·05% trypsin. The cells were washed 3 times in tissue culture medium and seeded into Falcon 3034 microplates. Cell attachment was usually completed after incubation for 3–4 h at 37°C, in humidified air + 5% $CO_2$. The plates were then inverted for 20 min and the medium drained off. Effector cell preparations suspended in tissue culture medium supplemented with 10 mmol/l Hepes buffer were added in 15 $\mu$l volumes to give the required effector : target cell ratios. Each effector cell preparation was tested at 2 ratios. A minimum of 12 wells was used for each parameter under test, including the effector cell-free medium control.

The plates were incubated at 37°C in humidified air + 5% $CO_2$ for 40–48 h (preliminary experiments showed that significant cytotoxicity was not routinely detected before this time). Experiments were terminated by inverting the plates for 20 min, draining off the medium, washing with phosphate buffered saline pH 7·2 and staining with May–Grünwald Giemsa.

*Calculation of cytotoxicity.*—The arithmetic mean of residual targets in wells which had contained patients' lymphoid cells was compared with that in wells having contained control donors' cells at equivalent ratios. The significance of differences were estimated by Student's *t* test with $P \leq 0{\cdot}05$.

Cytotoxicity is expressed as percent reduction $(1 - P/C) \times 100$; $P$ = mean residual targets after incubation with patient's lymphoid cells; C = mean residual targets after incubation with control donor's lymphoid cells; at equivalent concentrations.

Tumour specific cytotoxicity refers to a significant effect produced by a given donor's effector cells only on targets of a common histogenic origin. Nonspecific cytotoxicity is defined as an effect on targets of more than one histogenic origin.

### RESULTS

### *Composition of effector cell preparations*

Differential counts were made on the "purified" effector cell preparations from each blood donor. The pooled data for each patient group and clinical situation are shown in Table I. The significance of differences from normal healthy control donors was estimated by Student's *t* test. Patients with localized malignant melanoma (both ocular and skin) were found to have significantly more immature and myeloid precursor elements in the blood. Melanoma patients with widespread metastases had in addition increased numbers of eosinophils and significantly fewer recoverable lymphocytes. Patients clinically "cured" of melanoma showed no significant changes from normals. Among the clinical control donors patients with localized carcinoma of the bladder (TCC) also had increased numbers of immature

TABLE I.—*Cellular Content of Effector Preparations* (mean percentage ± SD)*

| Patient group | Lymphocytes | Neutrophils | Eosinophils | Basophils | Monocytes | Immature cells | Promyelocytes |
|---|---|---|---|---|---|---|---|
| Melanoma localized | 92·8±6·8 | 1·9±2·4 | 2·5±3·8 | 1·5±1·8 | 0·2±0·4 | 0·6±0·9 (<0·05)† | 0·6±1·1 (<0·05)† |
| Melanoma tumour-free | 94·7±3·0 | 1·4±2 | 1·7±1·6 | 1·7±1·7 | 0·17±0·2 | 0·2±0·5 | 0·07±0·16 |
| Melanoma metastases | 88·6±8 (0·05)† | 2·8±3 | 4·6±4·6 (0·05)† | 1·8±2 | 0·3±0·4 | 0·8±1·3 (<0·05)† | 1±1·5 (0·05)† |
| Ca bladder localized | 96·5±2 | 1±1 | 0·4±0·5 (0·02)† | 0·9±0·6 | 0·26±0·28 | 0·46±0·6 (0·05)† | 0·5±1·3 (0·05)† |
| Ca bladder tumour-free post radiation | 93·5±5·7 | 0·9±1·3 | 2·7±4·9 | 2·4±1·6 | 0·2±0·24 | 0·45±0·6 (0·05)† | 0·3±0·6 |
| Ca bladder metastases | 74±12 (<0·001)† | 5·6±5·7 (0·05)† | 15±16 (0·05)† | 2·3±2·5 | 0·2±0·4 | 0·7±0·7 (0·05)† | 2·7±5·6 (<0·05)† |
| Basal cell Ca | 95·7±4·6 | 2·4±3·8 | 0·7±1·1 | 0·8±0·5 | 0 (0·05)† | 0·2±0·25 | 0 |
| Normal healthy | 95±3 | 1·2±1·5 | 2·2±1·8 | 1·6±1·9 | 0·3±0·36 | 0·08±0·22 | 0·028±0·07 |

* Cells prepared as described in Materials and Methods.

† Significantly different from normal donors.

TABLE II.—*Nonspecific Cytotoxicity of Effector Cells from Patient A.R. with Metastatic Tumour*

| Target* | Effector | Effector : Target ratio | Surviving targets/well (mean ± SD) | % Reduction (5) | P |
|---|---|---|---|---|---|
| MEL-1 | (1) J 126 | 500 : 1 | 16±6 | — | |
| T.C.5 | | 250 : 1 | 24±10 | — | |
| | (2) S.M. autologous | 500 : 1 | 24±4 | 0 | |
| | | 250 : 1 | 26±6 | 0 | |
| | (3) A.R. | 500 : 1 | 4±2 | 75 | <0·001 |
| | | 250 : 1 | 4±2 | 83 | <0·001 |
| | (4) E.H. | 500 : 1 | 24±6 | 0 | |
| | | | 26±8 | 0 | |
| T24 | J 126 | 500 : 1 | 143±59 | — | |
| | | 250 : 1 | 184±90 | — | |
| | S.M. | 500 : 1 | 152±60 | 0 | |
| | | 250 : 1 | 201±51 | 0 | |
| | A.R. | 500 : 1 | 49±14 | 66 | <0·001 |
| | | 250 : 1 | 98±78 | 47 | 0·02 |
| | E.H. | 500 : 1 | 179±78 | 0 | |
| | | 250 : 1 | 267±49 | 0 | |

Medium control mean ± SD MEL-1, 28 ± 4 T24, 263 ± 72.

* MEL-1 derived from metastatic tumour of patient S.M.

(1) Untreated Ca prostate.

(2) S.M. malignant melanoma skin with lymph node and brain metastases.

(3) A.R. malignant melanoma skin with liver metastases.

(4) E.H. malignant melanoma skin tumour-free 6 years after preoperative radiotherapy and surgery.

(5) % Reduction estimated on J 126. Incubation time 45 h.

and myeloid precursor cells but decreased numbers of eosinophils in their effector cell preparations. Patients with metastatic TCC had in addition elevated numbers of eosinophils and neutrophils but much reduced numbers of recoverable lymphocytes. Tumour-free TCC patients post radiotherapy, differed from normal in having a still elevated level of immature cells. Patients with basal cell carcinoma differed from normals in having no detectable monocytes in their effector cell preparations. Patients with carcinoma of prostate or renal pelvis were too few for a separate statistical analysis; however, all patients with metastases showed a related picture to that observed in melanoma and TCC patients in the same stage.

*Nonspecific cytotoxicity*

A significant reduction in the number of surviving targets from more than one histogenic origin was observed with effector cells from 4/8 patients with metastatic malignant melanoma (this included one patient with amelanotic disease). This type of reaction is shown in Table II; the patient A.R. gave a significant effect on melanoma, nonmalignant bladder and TCC targets at 3 separate testings during a period of 1 year. Similarly, 4/6 patients with widespread metastatic TCC showed significant effects on both TCC and melanoma targets. All other melanoma patients with metastatic tumour showed no effect on any target tested; this is exemplified by S.M. in Table II who failed to respond against her own autologous cells (MEL-1) and against an allogeneic target T24. The correlation of nonspecific effects to metastatic disease could indicate a role for the contaminating non-lymphocytic elements in these effector preparations. Significant nonspecific cytotoxicity was observed consistently in only one other patient group; 3/6 untreated patients with basal cell carcinoma gave this type of reaction. Clearly from Table I this effect does not relate to the purity of the effector cell preparations or to therapy effects in this group.

TABLE IV.—*Effect of Preoperative Radiotherapy and Surgery on Tumour Specific Cytotoxicity of Effector Cells from Patient W.M. with Malignant Melanoma of Skin*

| Target | Effector | | Effector : Target ratio | Surviving targets/well (mean ± SD) | M.C.* | % Reduction (8) | *P* |
|---|---|---|---|---|---|---|---|
| T24 | (1) W.M. | 14.2.73 | 250 : 1 | 163±31 | | 0 | |
| | | untreated | 125 : 1 | 177±29 | | 0 | |
| | (2) J 145 | | 250 : 1 | 95±31 | 178±28 | 37 | <0·001 |
| | | | 125 : 1 | 142±26 | | 16 | <0·01 |
| | (3) J 145 A | | 250 : 1 | 150±20 | | — | |
| | | | 125 : 1 | 169±27 | | — | |
| MEL-1 | W.M. | | 250 : 1 | 130±25 | | 7 | NS |
| TC5 | | | 125 : 1 | 150±40 | | 6 | NS |
| | J 145 | | 250 : 1 | 150±30 | 150±30 | 0 | |
| | | | 125 : 1 | 170±50 | | 0 | |
| | J 145 A | | 250 : 1 | 140±19 | | — | |
| | | | 125 : 1 | 160±30 | | — | |
| T24 | W.M. | 15.2.73 | 250 : 1 | 76±10 | | 0 | |
| | | 24 h after | 125 : 1 | 80±10 | | 0 | |
| | (4) J.A. | 10,000 rad | 250 : 1 | 64±13 | 78±11 | 9 | NS |
| | | | 125 : 1 | 75±9 | | 1 | NS |
| | (5) J 146 A | | 250 : 1 | 70±11 | | — | |
| | | | 125 : 1 | 76±9 | | — | |
| MED-1 | W.M. | | 250 : 1 | 70±30 | | 52 | 0·001 |
| TC5 | | | 125 : 1 | 80±30 | | 49 | 0·001 |
| | J.A. | | 250 : 1 | 140±30 | 160±45 | 3 | NS |
| | | | 125 : 1 | 160±40 | | 2·5 | NS |
| | J 146 A | | 250 : 1 | 146±26 | | — | |
| | | | 125 : 1 | 156±40 | | — | |
| T24 | W.M. | 21.2.73 | 250 : 1 | 78±7 | 86±16 | 0 | |
| | | 5 days post | 125 : 1 | 90±22 | | 0 | |
| | (6) J 153 | excision | 250 : 1 | 74±18 | | — | |
| | | | 125 : 1 | 76±12 | | — | |
| MEL-1 | W.M. | | 250 : 1 | 33±12 | 29±11 | 0 | |
| TC5 | | | 125 : 1 | 31±6 | | 0 | |
| | J 153 | | 250 : 1 | 27±5 | | — | |
| | | | 125 : 1 | 29±5 | | — | |
| T24 | W.M. | 16.4.74 | 500 : 1 | 139±14 | 144±23 | 3·5 | NS |
| | | 1 year after | 250 : 1 | 150±46 | | — | |
| | (7) J 230 | excision | 500 : 1 | 144±30 | | — | |
| | | | 250 : 1 | 146±25 | | — | |
| MEL-1 | W.M. | | 500 : 1 | 63±19 | 68±16 | 0 | |
| TC26 | | | 250 : 1 | 84±15 | | 0 | |
| | J 230 | | 500 : 1 | 49±15 | | — | |
| | | | 250 : 1 | 55±10 | | — | |
| RPMI | W.M. | | 500 : 1 | 55±12 | 67±9 | 0 | |
| 7931 | | | 250 : 1 | 70±6 | | 0 | |
| | J 230 | | 500 : 1 | 50±11 | | — | |
| | | | 250 : 1 | 51±8 | | — | |

* M.C. Medium control targets incubated without lymphocytes.
(1) W.M. Malignant melanoma skin, stage I.
(2) J 145. TCC T1M1 untreated.
(3) J 145. A normal donor.
(4) J.A. Malignant melanoma skin, stage I. Four months after preoperative radiotherapy and tumour excision, clinically tumour-free.
(5) J 146. A normal donor.
(6) J 153. Basal cell ca, 1 month after local radiotherapy.
(7) J 230. Normal donor.
(8) % Reduction estimated on normal donors and J 153.
NS. Differences not significant.

TABLE IV.—*Effect of Preoperative Radiotherapy and Surgery on Tumour Specific Cytotoxicity of Effector Cells from Patient S.G. with Malignant Melanoma of Skin*

| Target | Effector | | Effector : Target ratio | Surviving targets/well (mean ± SD) | M.C.* | % Reduction (8) | *P* |
|---|---|---|---|---|---|---|---|
| RPMI 7931 | (1) S.G. | 30.4.73 | 250 : 1 | 8 ± 4 | | 43 | 0·01 |
| | | untreated | 125 : 1 | 13 ± 5 | | 0 | |
| | (2) J 174 | | 250 : 1 | 13 ± 5 | 22 ± 6 | 7 | NS |
| | | | 125 : 1 | 15 ± 4 | | 0 | |
| | (3) J 174 A | | 250 : 1 | 14 ± 6 | | — | |
| | | | 125 : 1 | 12 ± 5 | | — | |
| T24 | SG. | | 250 : 1 | 17 ± 4 | | 0 | |
| | | | 125 : 1 | 20 ± 6 | | 0 | |
| | J 174 | | 250 : 1 | 18 ± 4 | 20 ± 5 | 0 | |
| | | | 125 : 1 | 19 ± 4 | | 0 | |
| | J 174 A | | 250 : 1 | 16 ± 5 | | — | |
| | | | 125 : 1 | 18 ± 6 | | — | |
| RPMI 7931 | SG. | 3.5.71 | 250 : 1 | 17 ± 8 | | 26 | 0·05 |
| | | 48 h after | 150 : 1 | 28 ± 11 | | 0 | |
| | (4) J 176 | 10,000 rad | 250 : 1 | 21 ± 3 | 30 ± 10 | 9 | NS |
| | | | 150 : 1 | 26 ± 5 | | 4 | NS |
| | (5) J 176 A | | 250 : 1 | 20 ± 6 | | — | |
| | | | 150 : 1 | 27 ± 8 | | — | |
| T24 | SG. | | 250 : 1 | 26 ± 8 | | 4 | NS |
| | | | 125 : 1 | 30 ± 11 | | 0 | |
| | J 176 | | 250 : 1 | 17 ± 5 | 28 ± 1 | 237 | 0·05 |
| | | | 125 : 1 | 26 ± 4 | | 7 | NS |
| | J 176 A | | 250 : 1 | 27 ± 6 | | — | |
| | | | 125 : 1 | 28 ± 9 | | — | |
| RPMI 7931 | SG. | 14.5.73 | 500 : 1 | 63 ± 11 | | 0 | |
| | | 10 days post | 250 : 1 | 74 ± 9 | | 0 | |
| | (6) J 178 | excision | 500 : 1 | 62 ± 11 | 88 ± 9 | 0 | |
| | | | 250 : 1 | 74 ± 6 | | 0 | |
| | (7) J 179 A | | 500 : 1 | 60 ± 10 | | — | |
| | | | 250 : 1 | 70 ± 12 | | — | |
| MEL-1 TC20 | SG. | | 500 : 1 | 43 ± 5 | | 0 | |
| | | | 250 : 1 | 38 ± 7 | | 7 | NS |
| | J 178 | | 500 : 1 | 36 ± 8 | 32 ± 7 | 5 | NS |
| | | | 250 : 1 | 40 ± 7 | | 2 | NS |
| | J 178 A | | 500 : 1 | 38 ± 10 | | — | |
| | | | 250 : 1 | 41 ± 8 | | — | |
| T24 | SG. | | 500 : 1 | 20 ± 10 | | 9 | NS |
| | | | 250 : 1 | 24 ± 5 | | 8 | NS |
| | J 178 | | 500 : 1 | 25 ± 7 | 38 ± 16 | 0 | |
| | | | 250 : 1 | 24 ± 8 | | 8 | NS |
| | J 178 A | | 500 : 1 | 22 ± 8 | | — | |
| | | | 250 : 1 | 26 ± 10 | | — | |

* Medium control targets incubated without lymphocytes.

(1) SG. Malignant melanoma skin, stage I.
(2) J 174. TCC T3M4, tumour recurrence post radiotherapy.
(3) J 174 A. Normal donor.
(4) J 176. TCC T3M3, untreated.
(5) J 176 A. Normal donor.
(6) J 178. TCC T3M3, post radiotherapy, large tumour present.
(7) J 178 A. Normal donor.
(8) % Reduction estimated on normal donor in each test. Incubation time 48 h.
N.S. Differences not significant.

TABLE V.—*Tumour Specific Reactions of Skin Melanoma Patients Tested After Treatment*

| Target | Effector | Effector : Target ratio | Surviving targets/well (mean ± SD) | % Reduction | P |
|---|---|---|---|---|---|
| MEL-1 TC3 | (1) J 97 | 500 : 1 | 70±30 | — | — |
| | | 250 : 1 | 140±50 | — | — |
| | (2) E.A. | 500 : 1 | 30±15 | 57 | 0·01 |
| | | 250 : 1 | 110±40 | 21 | NS |
| | (3) H.B. | 500 : 1 | 90±30 | 0 | — |
| | | 250 : 1 | 130±30 | 7 | NS |
| | (4) S.K. | 500 : 1 | 0 | 100 | 0·001 |
| | | 250 : 1 | 70±20 | 50 | 0·01 |
| | (5) J 101 | 500 : 1 | 120±45 | 0 | — |
| | | 250 : 1 | 160±50 | 0 | — |
| | (6) J 102 | 500 : 1 | 60±20 | 14 | NS |
| | | 250 : 1 | 130±25 | 7 | NS |
| T24 | J 97 | 500 : 1 | 91±17 | — | — |
| | | 250 : 1 | 107±18 | — | — |
| | E.A. | 500 : 1 | 82±8 | 10 | NS |
| | | 250 : 1 | 93±13 | 13 | NS |
| | H.B. | 500 : 1 | 100±15 | 0 | — |
| | | 250 : 1 | 116±14 | 0 | — |
| | S.K. | 500 : 1 | 81±10 | 11 | NS |
| | | 250 : 1 | 91±11 | 15 | NS |
| | J 101 | 500 : 1 | 84±9 | 8 | NS |
| | | 250 : 1 | 94±19 | 12 | NS |
| | J 102 | 500 : 1 | 54±12 | 41 | 0·001 |
| | | 250 : 1 | 73±19 | 32 | 0·001 |
| RT4 | J 97 | 500 : 1 | 38±8 | — | — |
| | | 250 : 1 | 38±6 | — | — |
| | E.A. | 500 : 1 | 34±12 | 10·5 | NS |
| | | 250 : 1 | 34±8 | 10·5 | NS |
| | H.B. | 500 : 1 | 48±8 | 0 | — |
| | | 250 : 1 | 54±10 | 0 | — |
| | S.K. | 500 : 1 | 52±8 | 0 | — |
| | | 250 : 1 | 54±12 | 0 | — |
| | J 101 | 500 : 1 | 38±16 | 0 | — |
| | | 250 : 1 | 38±8 | 0 | — |
| | J 102 | 500 : 1 | 22±6 | 42 | 0·01 |
| | | 250 : 1 | 18±6 | 53 | 0·001 |

Medium control: Targets incubated without lymphocytes (mean ± SD) MEL-1, 160 ± 50; T24, 119 ± 20; RT4, 51±15.

(1) J 97. Tumour cutis capillitii, untreated.

(2) E.A. Malignant melanoma of skin stage I, 2 years after primary operation, 1 month after extended surgery, clinically tumour-free.

(3) H.B. Malignant melanoma of skin, stage III, 8 months after preoperative radiotherapy and surgery, metastases.

(4) S.K. Malignant melanoma of skin, stage I, 15 years after preoperative radiotherapy and surgery, 12 years after extended excision, clinically tumour-free.

(5) J 101. Basal cell Ca. untreated.

(6) J 102. TCC T2 M4, 2 months after 6·457 rad, tumour-free.

(7) % Reduction estimated on J 97. Incubation time 40 h.

N.S. Differences not significant.

*Tumour specific cytotoxicity*

This type of reaction was observed on both the primary culture MEL-1 and the cell line RPMI 7931 derived from metastatic malignant skin melanoma, by effector cells from certain donors with tumour of the same histogenic origin, with no effect on unrelated targets tested in parallel. Specific reactions were concurrently observed by effector cells from donors with TCC only on TCC targets with no effect on melanoma targets (Tables III, IV, V).

*Clinical observations in relation to CMI*

*Ocular tumour.*—The reactions obtained with effector cells from this group of patients are depicted in Table VII. Of the 5 patients tested before surgery, 2 showed melanoma specific reactions on targets derived from skin melanoma. Quantitative data on patient L.B. are given in Table VI. Significant effects were produced on both allogeneic melanoma targets, MEL-1 and RPMI 7931, with no effect on J82, a histogenically unrelated allogeneic target. In the same experiment, effectors from patient J.L. (stage I skin melanoma) showed the same range of specificity as LB's cells while effectors from S.O., a patient with metastatic skin melanoma, produced nonspecific effects.

*Effect of surgery on CMI in patients with ocular tumours.*—Only 2 patients were tested after surgery, at a 3–4 month interval; both failed to respond (Table VII). Patient F.E. had also been tested before operation and showed a nonspecific effect at that time. The only detectable difference in F.E.'s effector cell preparations was that 1% immature cells which were present before surgery were absent at test 4 months after treatment.

*Malignant melanoma of the skin.*—Among 3 patients tested before therapy (Table VIII) 1 case with stage I tumour showed a tumour specific reaction. The remaining patients with this diagnosis were all tested after treatment.

*Effect of preoperative radiotherapy and surgery on CMI.*—As shown in Table VIII, 3 patients were tested serially to assess the effect of this therapy. Changes in reactivity were seen in 2 cases—W.M. and S.G.—during and after therapy. Detailed quantitative data on the changes in tumour specific CMI in these patients are given in Tables III and IV. It can be seen that both patients showed a response during a 24–48 h interval after local irradiation to the tumour. (In the

TABLE VI.—*Tumour Specific Cytotoxicity of Effector Cells from Donors with Skin or Eye Melanoma*

| Target* | Effector | Effector : Target ratio | Surviving targets/well (mean ± SD) | % Reduction (5) | P |
|---|---|---|---|---|---|
| MEL-1 TC15 | (1) LB | 250 : 1 | 90±30 | 18 | 0·05 |
| | (2) JL | 250 : 1 | 70±30 | 36 | <0·001 |
| | (3) J 215 | 250 : 1 | 110±20 | — | — |
| | (4) SO | 250 : 1 | 100±25 | 9 | NS |
| RPMI 7931 | LB | 250 : 1 | 35±10 | 31 | 0·05 |
| | JL | 250 : 1 | 37±4 | 27·5 | 0·05 |
| | J 215 | 250 : 1 | 51±6 | — | — |
| | S.O. | 250 : 1 | 26±3 | 49 | <0·001 |
| J82 TC10 | LB | 250 : 1 | 63±12 | 0 | — |
| | JL | 250 : 1 | 63±17 | 0 | — |
| | J 215 | 250 : 1 | 58±13 | — | — |
| | S.O. | 250 : 1 | 48±12 | 17 | 0·05 |

* For Origin see Materials and Methods.
Medium control: targets incubated without lymphocytes (mean ± SD), MEL-1, 120 ± 4; RPMI 7931, 40 + 3; J 82, 68 + 4.
(1) LB. Melanosis oculi before operation.
(2) JL. Stage I skin melanoma, 3 weeks after operation, clinically tumour-free.
(3) J 215. TCC T3M4, 2 weeks after 4000 rad.
(4) S.O. Skin melanoma with hepatic and lymph node metastases.
(5) % Reduction estimated on control lymphocyte donor J 215; incubation time 48 h.
N.S. Difference not significant.

TABLE VII.—*Summary of CMI in Patients with Ocular Melanoma*

| Tumour location | Patient* | Surgery Before | Surgery After | Clinical situation |
|---|---|---|---|---|
| Epibulbar | S.T. | 0 (1) | NT | |
| Epibulbar** | L.B. | + | NT | |
| Intraocular | F.L. | NT | 3 months 0 | Tumour-free after surgery |
| Intraocular | F.E. | Nonspecific | 4 months 0 | Tumour-free after surgery |
| Intraocular | M.A. | 0 | NT | |
| Intraocular | F.S. | + | NT | |
| Incidence tumour specific CMI | | 2/5 | 0/2 | |

(1) 8 years after local resections and post-operative radiotherapy.
* For clinical details see Appendix Table II.
** Epibulbar location: melanosis in the conjunctiva.
NT Not tested.

TABLE VIII.—*Summary of CMI in Patients with Malignant Melanoma of the Skin Tested Before and After Treatment by Preoperative Radiotherapy and Surgery*

| Tumour stage | Patient | Untreated | After radiotherapy | Post surgery | Clinical situation post therapy |
|---|---|---|---|---|---|
| I | W.M. | 0 | 24 h+ | 5 days 0 | Tumour-free |
| | | | | 1 year 0 | Tumour-free |
| | G.G. | 0 | NT | 4 months 0 | Tumour-free |
| | S.G. | + | 48 h+ | 10 days 0 | Tumour-free |
| Incidence of tumour specific CMI | | 1/3 | 2/2 | 0/3 | |

NT: Not tested.

case W.M. no reaction had been detectable before therapy.) The 3 patients were tested after surgery and all showed no response; only W.M. was tested more than once after surgery.

A further 10 patients who received preoperative radiotherapy and surgery were available for testing only post therapy. The observations on this group are summarized in Table IX. Patients in stages I and II were clinically tumour-free, but a single case H.B. with stage III disease had distant metastases. As shown in Table IX, only 1 patient, S.K., in this series gave tumour specific reactions. Clinically, S.K. appears tumour-free 13·5 years after therapy. Specific responses were detected in tests performed 12 and 13 years after therapy. Quantitative details of one test on S.K. are given in Table V. Experimental details on patients E.H. and J.A. are given in Tables II and III respectively. Patient H.B. was tested in the same experiment as S.K. and showed no response. Clearly patients in this group should undergo further serial testing to access possible clinical significance of tumour specific CMI.

*Effect of local tumour excision on CMI.*—No patients were tested both before and after local surgery, but 12 cases were available after treatment. These are summarized in Table X. All patients with a stage I diagnosis were clinically tumour-free post surgery. Tumour specific reactions were detected in the 2 patients tested 3–4 weeks after surgery. (Details of patient E.A. are given in Table V.) With 1 exception (patient G.A.), all the remaining cases with stage I tumour tested at longer time intervals (5 months–3 years) after surgery showed no reaction. As with melanoma patients tested after

TABLE IX.—*Summary of CMI in Patients with Skin Melanoma Tested After Preoperative Radiotherapy and Surgery*

| Tumour stage | Patient | Time after treatment | CMI | Clinical situation after treatment |
|---|---|---|---|---|
| I | M.A. | 4 months | 0 | Tumour-free |
| | J.Ar. | 4 months | 0 | Tumour-free |
| | | 1 year | | |
| | J.La. | 6 months | 0 | Tumour-free |
| | G.A.S. | 7 months | 0 | Tumour-free |
| | W.E. | 4 years | 0 | Tumour-free |
| | L.S. | 5 years | 0 | Tumour-free |
| | E.H. | 6 years | 0 | Tumour-free |
| | S.K. | 12 years | + | Tumour-free |
| | | 13 years | + | Tumour-free |
| II | F.E. | 2 months* | 0 | Tumour-free |
| III | H.B. | 8 months | 0 | Metastases |
| Incidence of tumour specific CMI | | | 1/10 | |

* Patient received post-operative radiotherapy 1 month after surgery.

TABLE X.—*Summary of CMI in Patients with Skin Melanoma Tested After Surgery*

| Tumour stage | Patient | Time after operation | CMI | Clinical situation after treatment |
|---|---|---|---|---|
| I | J.L. | 3 weeks | + | Tumour-free |
| | E.A. | 4 weeks | + | Tumour-free |
| | N.E. | 5 months | 0 | Tumour-free |
| | J.A. | 1 year | 0 | Tumour-free |
| | G.A. | 2 years | + | Tumour-free |
| | | 3 years | 0 | Tumour-free |
| | A.E. | 2 years | 0 | Tumour-free |
| | | 3 years | 0 | Tumour-free |
| | L.A.M. | 2 years | 0 | Tumour-free |
| | O.A.M. | 3 years | 0 | Tumour-free |
| II | P.G. | 1 week | Nonspecific | Metastases |
| | S.M. | 1 month | 0 | Metastases |
| III | S.O. | 3 weeks | Nonspecific | Metastases |
| | | 6 months | 0 | |
| | A.R. | 14 days* | Nonspecific | Metastases |
| | | 1 month | Nonspecific | |
| | | 1 year | Nonspecific | |
| Incidence of tumour specific CMI | | | 3/12 | |

* Tested before palliative radiotherapy.

other types of therapy, periodic retesting is required to define the clinical significance of this response. Patients in this group with higher stage tumours all had metastases at time of test. Non-reactive patient S.M. is presented in Table II. The nonspecific effect produced by A.R.'s and S.O.'s cells are quantitated in Tables II and VI.

*Systemic effects of local radiotherapy.*—Three patients with stage II and III melanoma were given palliative radiotherapy in total doses of 4000–4960 rad to the draining lymph nodes. The resulting effects on the detectable numbers of peripheral lymphoid cells are shown in Table V Appendix. A drastic lymphopenia was apparent within 48 h of cessation of therapy. Similarly, effects have been observed after local radiotherapy in patients with TCC to the region of the urinary bladder, the duration of which also depended on the clinical situation (whether tumour-free or not) of the patient post therapy. (B. Unsgaard, to be published.) It should be noted that these patients were treated palliatively and all succumbed to widespread metastases (Tables II, VIII, X).

### DISCUSSION

These results demonstrate that the incidence and type of cytotoxicity produced by effector cell preparations from melanoma patients are influenced by tumour burden and therapeutic intervention. The method of effector cell preparation was shown to yield a heterogeneous population of lymphoid and myeloid precursor cells from certain patient groups, particularly those with metastatic tumour. Detailed analyses of the composition of effector cell preparations used in *in vitro* assays for CMI from patients in different clinical situations have not previously been reported. The high incidence of nonspecific effects on histogenically diverse targets observed with preparations from patients with metastases could implicate non-lymphocytic cells in these effects. Neutrophils, eosinophils and myeloid precursor cells are known to be rich in hydrolytic enzymes. Such enzymes have been shown to exert a powerful cytolytic effect on a variety of mammalian target cells *in vitro* (Edelson and Cohn, 1973). However, effector cell preparations from patients with basal cell carcinoma also showed a significant incidence of nonspecific effects despite no obvious non-lymphocytic contamination. Elucidation of the role and target specificity range of different effector cell populations in *in vitro* cytotoxicity assays will require the procurement of more homogeneous populations of effector cell types and standardization of preparative procedures.

Tumour specific cytotoxicity could be quantitated in certain patients with ocular and skin melanomata. A long-term cell line and a primary culture derived from metastatic skin melanomata were compared as allogeneic targets and found to give qualitatively comparable results. It has previously been reported that sera from some patients with ocular melanoma contain antibodies which react specifically with skin melanoma target cells (Nairn *et al.*, 1972; Federman, Lewis and Clark, 1974). Our data demonstrate cross reactivity also at the cellular level.

The incidence of tumour specific CMI detected in this series of melanoma patients contrasts sharply with that reported for patients with the same diagnosis and in similar clinical situations by Hellström *et al.* (1971, 1973*a*, *b*). These authors found that the majority of clinically cured patients had tumour specific CMI during a 1–2 year observation period post surgery. In a similar group of patients we have found tumour specific CMI a rare phenomenon. Fossati *et al.* (1971) reported a high incidence of tumour specific CMI in patients 1–6 months after surgery; in the present series we observed reactivity mainly during a 3–4 week interval after surgery. Similar results have been reported by Nairn *et al.* (1972) who found no reactivity in melanoma patients tested 2 months post surgery.

Using similar *in vitro* assays, several authors have reported a diminution of tumour specific CMI in some melanoma patients with disseminated disease (De Vries *et al.*, 1972; Hellström *et al.*, 1973*a*, *b*; Heppner *et al.*, 1973). Currie *et al.* (1971) reported a correlation in the incidence of CMI and extent of disease; however, this was not apparent after more extensive washing of effector cell preparations (Currie, 1973). Cochran *et al.* (1973) using a leucocyte migration inhibition assay, found correlations with the stage of disease. In the present series, tumour specific reactions were detected only in patients with localized disease while patients with disseminated melanoma were either nonreactive or showed nonspecific cytotoxicity. It should be noted that all effector cell preparations tested were washed a minimum of 6 times during preparation. The incidence of tumour specific CMI in patients with TCC of the bladder has been shown to correlate with extent of disease, patients with localized tumours showing the highest incidence (O'Toole *et al.*, 1972*a*, *b*, 1973*a*).

Comparisons of the clinical significance of the incidence of CMI in patients with melanoma, reported by different authors, cannot at present be made due to the disparity in methods of effector cell preparation, effector : target cell ratio and probable heterogeneity.

Significant clinical correlations have, however, been reported between the level of specific serum blocking factor (SBF) for CMI, *in vitro* and extent of disease (Hellström *et al.*, 1973*b*). *In vitro* quantitation of tumour specific CMI and its relation to the serum levels of SBF remain to be determined. However, a correlation between the level of tumour specific antibodies and the extent of disease has been documented in patients with malignant melanoma (Morton *et al.*, 1968, 1971; Lewis *et al.*, 1969).

Local radiotherapy and surgery have been shown to modify tumour specific CMI in patients with TCC of the bladder (O'Toole *et al.*, 1972*a*, *b*, 1973*a*). The maintenance of CMI in these patients was shown to be dependent on the presence of critical amounts of tumour material in the body. Successful removal of tumour by surgery led to the disappearance of specific CMI within 2–3 weeks post surgery. The majority of tumour-free melanoma patients tested at intervals greater than 1 month post surgery in this series also lacked tumour specific CMI. Radiotherapy in patients with TCC could induce a specific CMI in some cases which were previously nonreactive. This was observed also in a single melanoma case in the present series.

Patients with TCC given preoperative radiotherapy were shown to maintain tumour specific CMI for a period of several months after surgery (O'Toole *et al.*, 1972*b*, 1973*a*). This effect was not observed in the melanoma patients given preoperative radiotherapy. Irradiation technique, duration, time interval between radiotherapy and surgery and the actual tumour volume irradiated could determine these differences.

Tumour specific CMI has not been observed in patients with TCC who have remained tumour-free during a 1–12 year period after radiotherapy. However, responses were detected in patients who developed local recurrences after therapy (O'Toole *et al.*, 1973*a*). The relevance of tumour specific CMI to the aetiology of disease in the 2 melanoma patients who showed tumour specific reactions 2 and 12–13 years after therapy respectively, remains to be elucidated.

Patients with metastatic melanoma given palliative local radiotherapy to the axillary or inguinal region in doses of 4000–4960 rad were seen to develop pronounced lymphopenia when tested 2–14 days after treatment. Similar effects have been observed after local radiotherapy (Thomas *et al.*, 1971; Stjernswärd *et al.*, 1973; Chee, Ilberry and Rickinson, 1974) to other areas of the body.

We wish to thank Mrs Anna-Greta Göransson and Mrs Margareta Karlsson for excellent technical assistance. The work was supported from Grant no. 73 : 213 from the Swedish Cancer Society and The Jönköping Cancer Fund for Clinical Research.

### APPENDIX

Therapy used for malignant melanoma:

*Surgery.*—The primary tumour was excised in patients with stage I skin melanoma. Stage II patients were treated by local excision of the primary and dissection of the draining lymph nodes. For stage III the primary tumour was excised to reduce tumour burden, and then cytostatics and/or external radiation were given palliatively.

*Radiotherapy.*—Stage I malignant melanoma of the skin is treated at this hospital by preoperative radiotherapy. This was given with a Dermopan 2 unit operated at 10–50 kV, 25 mA, either without filter or with 0·3–1 mm Al. The total skin dose was 10,000 rad, given with a margin, followed 24–120 h later by excision of the treated area with about 1 cm margin.

Therapy and clinical details of patients with transitional cell carcinoma of the urinary bladder:

Patients receiving radiotherapy were given either $^{60}Co$ teletherapy or betatron 18 MeV roentgen. Two patients received 4000–4200 rad preoperatively and 12 had full dose treatment of 6270–6725 rad. Patients treated surgically had transurethral resection only or total cystectomy 1 month after radiotherapy. The clinical staging of

APPENDIX TABLE I.—*Total Patients with Melanoma Tested*

| Site of origin | Female | Male | Total |
|---|---|---|---|
| Eye | 3 | 3 | 6 |
| Head and neck | 3 | | 3 |
| Upper limb | 2 | 2 | 4 |
| Trunk | 3 | 6 | 9 |
| Lower limb | 6 | 2 | 8 |
| Vulva | 1 | | 1 |
| Unknown* | | 2 | 2 |
| Total | 18 | 15 | 33 |

* Patients with generalized tumour process. Site of primary tumour unknown.

APPENDIX TABLE II.—*Summary of Clinical Data on Patients with Malignant Melanoma or Melanosis of the Eye*

| Patient | Age | Sex | | Situation at time of test |
|---|---|---|---|---|
| S.T. | 58 | Female | Epibulbar tumour. 14 years history of a malignant melanoma in the conjunctiva. Several recurrences with local excisions. At the time of test local recurrence with 2 tumours, 4 mm in diameter, surrounded by small satellites | Before enucleation of the eye. |
| L.B. | 46 | Male | Epibulbar location. Several years duration of melanosis in the conjunctiva | Untreated. |
| F.L. | 73 | Male | Intraocular tumour. 5 years history of an intraocular tumour in the right eye. Enucleation was performed. PAD showed a malignant melanoma, 10 mm in diameter, in the choroid with detachment of the retina. The tumour was composed of large pleomorphic cells with large ovoid nuclei. Few mitoses. Pigment content was sparse | 3 months after enucleation. Clinically estimated as tumour-free. |
| F.E. | 71 | Female | Intraocular tumour. 2 months history of decreased sight of left eye. Intraocular tumour was revealed. Enucleation of the eye. PAD showed malignant melanoma, 9 mm in diameter and 4 mm in height, growing in the choroid, partly infiltrating into the sclera, but without breaking through the sclera. The tumour was composed of slender spindle shaped cells with fusiform nuclei. Abundance of melanin containing macrophages | The first test was made before enucleation. The second test 4 months after enucleation. Clinically estimated as tumour-free. |
| M.A. | 8 | Female | Intraocular tumour. 6 months history of a growing brown pigmented spot in the iris. Partial iridectomy. PAD showed malignant melanoma with doubtful radicality. Removal of the eye. PAD showed local recurrence of malignant melanoma in the iris and local metastases to the anterior chamber | Before the primary operation. |
| F.S. | 74 | Male | Intraocular tumour. Malignant melanoma of the iris | Untreated. |

Appendix Table III.—*Number and Clinical Stage Distribution of Patients with Malignant Melanoma in the Skin*

| Classification | | No. of patients |
|---|---|---|
| Stage I | Localized melanoma confined to the skin. Local recurrences and nearby deposits in cutaneous lymphatics are included | 18 |
| Stage II | Cases with regional lymph node metastases confined to one gland station only | 4 |
| Stage III | Metastatic involvement of 2 or more groups of glands, and cases with distant metastases evincing generalized tumour process | 2 |

TCC (Table IV) is that proposed by UICC (1963); and the histological grading used was according to Bergkvist *et al.* (1965). The 29 patients with TCC were distributed according to tumour grade as follows: grade I, 2 cases; grade II, 8 cases; grade III, 13 cases and grade IV, 6 cases.

Age distribution of patients and controls used in this study:

Patients with malignant melanoma of the skin were aged 28–85 (mean 53) years. Those with ocular tumours 8–74 (mean 55) years. Patients with basal cell carcinoma 56–85 (mean 67) years. Patients with TCC 57–82 (mean 67) years. Normal controls 20–59 (mean 38) years.

Appendix Table IV.—*Clinical Controls*

| | | Tested after | | | |
|---|---|---|---|---|---|
| | | Irradiation | Surgery | Irradiation | Surgery |
| | | Clinical situation: | | | |
| Diagnosis | Untreated | Tumour-free | | Tumour present | |
| TCC tumour stage | | | | | |
| T1 | 2 | | | | |
| T2* | 4 | 9 | 3 | 2 | |
| T3† | 7 | 5 | | 6 | |
| T4‡ | | | | 2 | |
| Transitional cell ca (TCC) Total | 13 | 14 | 3 | 10 | |
| Ca renal pelvis | | | 2 | | 1 |
| Ca prostate | 1 | | | 1 | |
| Ca rectum with metastases urinary bladder | 1 | | | | |
| Ca skin | 6§ | 1 | | | |
| Histiocytoma skin | 1 | | | | |
| Normal healthy | 10 | | | | |

* 2 patients were tested 3 times after irradiation.
1 patient was tested before and after irradiation.
1 patient was tested during irradiation and after cystectomy.

† 3 patients were tested twice after irradiation.
1 patient was tested before and after irradiation.

‡ This patient was tested twice.

§ 1 patient tested before and after local radiotherapy.

APPENDIX TABLE V.—*Effect of Local Radiotherapy on Peripheral Blood Leucocyte and Lymphocyte Counts in Patients with Malignant Melanoma of the Skin*

| Tumour stage | | Tumour site | Time of test | Total leucocytes/mm³ | Total lymphocytes/mm³ |
|---|---|---|---|---|---|
| II | S.M., 49 years, female | Lower limb | Untreated | 5200 | 2652 |
| | | | 48 h after 4960 rad given over 58 days to inguinal region | 4100 | 1107 |
| | | | 2 weeks post irradiation | 3100 | 372 |
| II | P.G., 49 years, male | Truncus | Untreated | 9400 | 1974 |
| | | | 48 h after 4000 rad given over 21 days to the axilla | 5200 | 260 |
| III | A.R., 53 years, male | Truncus | Untreated | 9500 | 3610 |
| | | | 48 h after 4000 rad given over 15 days to the axilla | 5400 | 594 |

### REFERENCES

BERGKVIST, A., LJUNGQUIST, A. & MOBERGER, G. (1965) Classification of Bladder Tumours Based on the Cellular Pattern. *Acta chir. scand.*, **130**, 371.

BOYLE, W. (1968) An Extension of the $^{51}$Cr-release Assay for the Estimation of Mouse Cytotoxins. *Transplantation*, **6**, 761.

BUBENIK, J., BARESOVA, M., VIKLICKY, V., JACOUBKOVA, J., SAINEROVA, H. & DONNER, J. (1973) Established Cell Line of Urinary Bladder Carcinoma (T24) Containing Tumour Specific Antigen. *Int. J. Cancer*, **11**, 765.

CHEE, C. A., ILBERY, L. T. & RICKINSON, A. R. (1974) Depression of Lymphocyte Replicating Ability in Radiotherapy Patients. *Br. J. Radiol.*, **47**, 37.

COCHRAN, A. J., MACKIE, R. N., THOMAS, C. E., GRANT, R. N., CAMERON-MOWAT, D. E. & SPILG, W. G. S. (1973) Cellular Immunity to Breast Carcinoma and Malignant Melanoma. In *Immunology of Malignancy*. Ed. M. Moore, N. W. Nesbet and Mary V. Haigh. *Br. J. Cancer*, **28**, Suppl. I, 77.

COULSON, A. S. & CHALMERS, D. G. (1964) Separation of Viable Lymphocytes from Human Blood. *Lancet*, i, 468.

CURRIE, G. (1973) The Role of Circulating Antigen as an Inhibitor of Tumour Immunity in Man. In *Immunology of Malignancy*. Ed. M. Moore N. W. Nisbet and Mary V. Haigh. *Br. J. Cancer*, **28**, Suppl. I, 153.

CURRIE, G. A., LEJEUNE, F. & FAIRLEY, G. H. (1971) Immunization with Irradiated Tumour Cells and Specific Lymphocyte Cytotoxicity in Malignant Melanoma. *Br. med. J.*, ii, 305.

DORÉ, D. F. & BALFOUR, B. N. (1965) A Device for Preparing Cell Spreads. *Immunology*, **9**, 403.

DE VRIES, J. E., RÜMKE, P. & BERNHEIM, J. L. (1972) Cytotoxic Lymphocytes in Melanoma Patients. *Int. J. Cancer*, **9**, 567.

EDELSON, P. J. & COHN, Z. A. (1973) Peroxidase Mediated Mammalian Cell Cytotoxicity. *J. exp. Med.*, **138**, 318.

FEDERMAN, J. L., LEWIS, M. G. & CLARK, W. H. (1974) Tumor Associated Antibodies to Ocular and Cutaneous Malignant Melanomas: Negative Interaction with Normal Choroidal Melanocytes. *J. natn. Cancer Inst.*, **52**, 587.

FOSSATI, G., COLNAGHI, M. I., DELLA PORTA, G., CASCINELLI, N. & VERONESI, U. (1971) Cellular and Humoral Immunity Against Human Malignant Melanoma. *Int. J. Cancer*, **8**, 344.

GREENWALT, J. T., GAJEWSKI, M. & MCKENNA, J. C. (1962) A New Method for Preparing Buffy-coat-poor Blood. *Transfusion*, **2**, 221.

HELLSTRÖM, I., HELLSTRÖM, K. E., SJÖGREN, H. O. & WARNER, G. A. (1971) Demonstration of Cell-mediated Immunity to Human Neoplasms of Various Histological Types. *Int. J. Cancer*, **7**, 1.

HELLSTRÖM, I. & HELLSTRÖM, K. E. (1973*a*) Some Recent Studies on Cellular Immunity to Human Melanomas. *Fedn Proc.*, **32**, 156.

HELLSTRÖM, I., WARNER, G. A., HELLSTRÖM, K. E. & SJÖGREN, H. O. (1973*b*) Sequential Studies of Cell-mediated Tumour Immunity and Blocking Serum Acitivity in Ten Patients with Malignant Melanoma. *Int. J. Cancer*, **11**, 280.

HEPPNER, G. H., STOLBACH, L., BYRNE, M., CUMMINGS, F. J., MCDONOUGH, E. & CALABRESI, P. (1973) Cell-mediated and Serum Blocking Reactivity to Tumour Antigens in Patients with Malignant Melanoma. *Int. J. Cancer*, **11**, 245.

JEHN, U. W., NATHANSON, L. & SCHWARTZ, R. S. (1970) *In vitro* Lymphocyte Stimulation by a Soluble Antigen from Malignant Melanoma. *New Engl. J. Med.*, **283**, 329.

LEWIS, M. G., IKONOPISOV, R. L., NAIRN, R. C., PHILLIPS, T. M., FAIRLEY, H. G., BODENHAM, D. C. & ALEXANDER, P. (1969) Tumour Specific Antibodies in Human Malignant Melanoma and their Relationship to the Extent of the Disease. *Br. med. J.*, iii, 547.

MORTON, D. L., MALMGREN, R. A., HOLMES, E. C. & KETCHAM, A. S. (1968) Demonstration of Antibodies Against Human Malignant Melanoma by Immunofluorescence. *Surgery, St Louis*, **64**, 233.

MORTON, D. L., EILBER, F. R. & MALMGREN, R. A. (1971) Immune Factors in Human Cancer: Malignant Melanomas, Skeletal and Soft Tissue Sarcomas. *Prog. exp. Tumor Res.*, **14**, 25.

NAIRN, R. C., NIND, A. P. P., GULI, E. P. G., DAVIES, D. J., LITTLE, J. H., DAVIS, N. C. & WHITEHEAD, R. H. (1972) Anti-tumour Immunoreactivity in Patients with Malignant Melanoma. *Med. J. Aust.*, **1**, 397.

O'TOOLE, C. (1973) Standardization of the Microcytotoxicity assay for Cell Mediated Immunity. *Natn. Cancer Inst. Monog.*, **37**, 19.

O'TOOLE, C., PERLMANN, P., UNSGAARD, B. MOBERGER, G. & EDSMYR, F. (1972*a*) Cellular Immunity to Human Urinary Bladder Carcinoma. I. Correlation to Clinical Stage and Radiotherapy. *Int. J. Cancer*, **10**, 77.

O'TOOLE, C., PERLMANN, P., UNSGAARD, B., ALMGARD, L. E., JOHANSSON, B., MOBERGER, G. & EDSMYR, F. (1972*b*) Cellular Immunity to Human Urinary Bladder Carcinoma. II Effect of Surgery and Preoperative Irradiation. *Int. J. Cancer*, **10**, 92.

O'TOOLE, C., UNSGAARD, B., ALMGARD, L. E. & JOHANSSON, B. (1973*a*) The Cellular Immune Response to Carcinoma of the Urinary Bladder: Correlation to Clinical Stage and Treatment. In *Immunology of Malignancy*. Ed. M. Moore, N. W. Nisbet and Mary V. Haigh. *Br. J. Cancer*, **28**, Suppl. I, 266.

O'TOOLE, C., PERLMANN, P., WIGZELL, H., UNSGAARD, B. & ZETTERLUND, C. G. (1973*b*) Lymphocyte Cytotoxicity in Bladder Cancer. No Requirement for Thymus Derived Effector Cells? *Lancet*, i, 1085.

O'TOOLE, C., STEJSKAL, V., PERLMANN, P. & KARLSSON, M. (1974) Lymphoid Cells Mediating Tumour Specific Cytotoxicity to Carcinoma of the Urinary Bladder; Separation of the Effector Population Using a Surface Marker. *J. exp. Med.*, **139**, 457.

RIGBY, C. C. & FRANKS, L. M. (1970) A Human Tissue Culture Cell Line from Transitional Cell Tumour of the Urinary Bladder: Growth, Chromosome Pattern and Ultrastructure. *Br. J. Cancer*, **24**, 746.

STJERNSWÄRD, J., VANKY, F., JONDAL, M., WIGZELL, H. & SEALY, R. (1973) Lymphopenia and Change in Distribution of Human B and T Lymphocytes in Peripheral Blood Induced by Irradiation for Mammary Carcinoma. *Lancet*, i, 1352.

TAKASUGI, M. & KLEIN, E. (1970) A Microassay for Cell-mediated Immunity. *Transplantation*, **9**, 219.

THOMAS, J. W., COY, P., LEWIS, H. S. & YUEN, A. (1971) Effect of Therapeutic Irradiation on Lymphocyte Transformation in Lung Cancer. *Cancer, N.Y.*, **27**, 1046.

UICC (Union Internationale Contre Le Cancer) (1963) *Cancer of the Urinary Bladder*. Basle and New York: Karger.